# [μ-6,9-Bis(carboxylatomethyl)-3,12-bis(car­boxymethyl)-3,6,9,12-tetraaza­tetradecanedioato]bis­[aqua­cobalt(II)] tetra­hydrate

**DOI:** 10.1107/S1600536813000196

**Published:** 2013-01-12

**Authors:** Qi-feng Qian, Jin-hui Wu, Jin-liang Qian

**Affiliations:** a Xuchang Senior School, Xuchang 461000, People’s Republic of China; b Zhile Second Middle School, Xuchang 461232, People’s Republic of China

## Abstract

The binuclear title complex, [Co_2_(C_18_H_26_N_4_O_12_)(H_2_O)_2_]·4H_2_O, lies about a centre of inversion, the Co^II^ atom being coordinated in a distorted octa­hedral arrangement defined by one water mol­ecule and N_2_O_3_ donors derived from one end of a 6,9-bis(carboxylatomethyl)-3,12-bis(car­boxy­methyl)-3,6,9,12-tetraaza­tetradecanedioate (H_2_TTHA^4−^) tetra­anion. In the crystal, numerous O—H⋯O hydrogen bonds link the mol­ecules into a three-dimensional network.

## Related literature

For related coordination complexes of species derived from triethyl­ene­tetra­minehexa­acetic acid, see: Ouyang *et al.* (2007 ▶); Xu *et al.* (2008 ▶). For a related structure, see: Song *et al.* (2003 ▶).
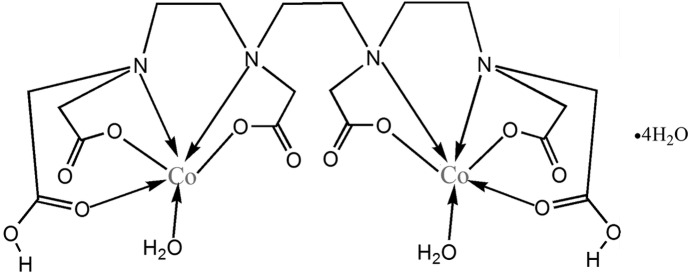


## Experimental

### 

#### Crystal data

[Co_2_(C_18_H_26_N_4_O_12_)(H_2_O)_2_]·4H_2_O
*M* *_r_* = 716.38
Triclinic, 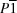

*a* = 7.0972 (15) Å
*b* = 8.7025 (19) Å
*c* = 11.968 (3) Å
α = 104.238 (4)°
β = 100.986 (3)°
γ = 100.425 (4)°
*V* = 682.9 (3) Å^3^
*Z* = 1
Mo *K*α radiation
μ = 1.31 mm^−1^
*T* = 292 K
0.10 × 0.08 × 0.05 mm

#### Data collection

Bruker SMART APEX CCD diffractometer
Absorption correction: multi-scan (*SADABS*; Bruker, 2001 ▶) *T*_min_ = 0.881, *T*_max_ = 0.938
8012 measured reflections
3099 independent reflections
2182 reflections with *I* > 2σ(*I*)
*R*_int_ = 0.097

#### Refinement

*R*[*F* ^2^ > 2σ(*F* ^2^)] = 0.053
*wR*(*F* ^2^) = 0.117
*S* = 0.94
3099 reflections
190 parameters
H-atom parameters constrained
Δρ_max_ = 0.61 e Å^−3^
Δρ_min_ = −0.53 e Å^−3^

### 

Data collection: *SMART* (Bruker, 2001 ▶); cell refinement: *SAINT* (Bruker, 2001 ▶); data reduction: *SAINT*; program(s) used to solve structure: *SHELXS97* (Sheldrick, 2008 ▶); program(s) used to refine structure: *SHELXL97* (Sheldrick, 2008 ▶); molecular graphics: *PLATON* (Spek, 2009 ▶); software used to prepare material for publication: *SHELXL97*.

## Supplementary Material

Crystal structure: contains datablock(s) global, I. DOI: 10.1107/S1600536813000196/tk5188sup1.cif

Structure factors: contains datablock(s) I. DOI: 10.1107/S1600536813000196/tk5188Isup2.hkl

Additional supplementary materials:  crystallographic information; 3D view; checkCIF report

## Figures and Tables

**Table 1:** Hydrogen-bond geometry (Å, °)

*D*—H⋯*A*	*D*—H	H⋯*A*	*D*⋯*A*	*D*—H⋯*A*
O1—H1⋯O1^i^	0.82	1.68	2.483 (5)	168
O5—H5⋯O5^ii^	0.82	1.67	2.481 (5)	170
O7—H7*A*⋯O8	0.82	1.84	2.616 (4)	157
O7—H7*B*⋯O4^iii^	0.82	1.95	2.732 (4)	159
O8—H8*D*⋯O3^iii^	0.82	2.37	2.745 (5)	109
O8—H8*C*⋯O9	0.82	2.15	2.719 (5)	127
O9—H9*A*⋯O2^iv^	0.82	2.48	3.067 (4)	130
O9—H9*A*⋯O6^iv^	0.82	2.45	3.217 (5)	157

Symmetry codes: (i) 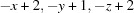; (ii) 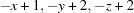; (iii) 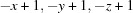; (iv) 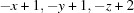.

**Fig. 1.**
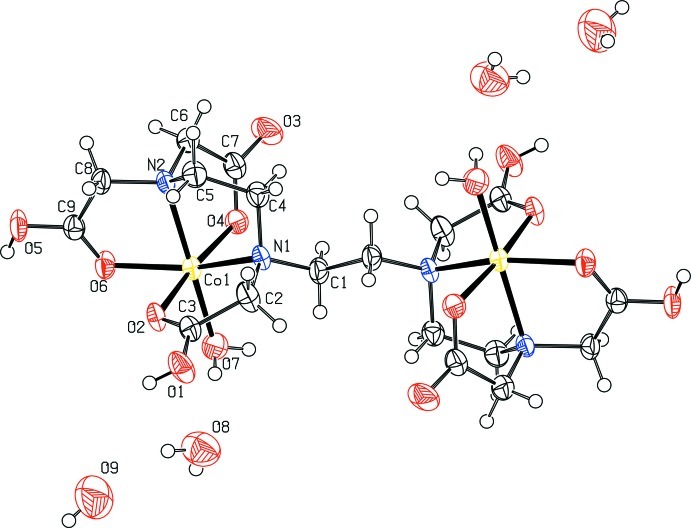
The molecular structures of the components of (I) with displacement ellipsoids drawn at the 50% probability level. Unlabelled atoms are related by the symmetry operation: 2-x, 1-y, 1-z.

**Fig. 2.**
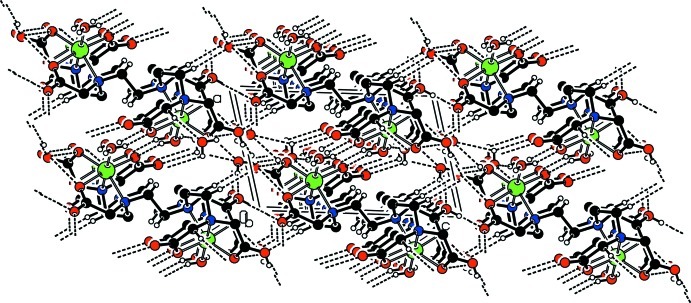
Part of the crystal structure of (I), showing the formation of a three-dimensional network by hydrogen bonds (dashed lines). Hydrogen atoms not involved in hydrogen bonding have been omitted for clarity.

## supplementary crystallographic information

### Comment

H_6_TTHA (triethylenetetraminehexaacetic acid) is a multicarboxyl ligand with ten potential coordinating sites and plays an important role in the self-assembly of various functional materials (Xu *et al.*, 2008; Ouyang *et al.*, 2007). In an effort to explore new enzyme-mimics involved with cobalt(II) and poly-carboxyl-group ligands, we have synthesized and crystallized the title complex, [Co_2_(H_2_TTHA)(H_2_O)_2_].4H_2_O, (I), in water under ambient conditions *via* the reaction of Co(OH)_2_ and H_6_TTHA. Herein, we report its crystal structure.

The asymmetric unit of (I) comprises half a neutral [Co_2_(H_2_TTHA)(H_2_O)_2_] binuclear unit and two solvent water molecules (Fig. 1). The binuclear [Co_2_(H_2_TTHA)(H_2_O)_2_] is centrosymmetric with the midpoint of the ethylene C—C bond on an inversion centre. Each Co^II^ ion has a distorted octahedral geometry and is bonded to two N atoms and three carboxylate-O atoms from half of the H_2_TTHA^4-^ ligand, as well as a water molecule. The Co1—N1 and Co1—N2 bond lengths are 2.216 (3) and 2.134 (3) Å, respectively, and the Co—O bond lengths range from 2.031 (3) to 2.120 (3) Å which are similar to those in its analogous structure (Song *et al.*, 2003)

Analysis indicates (Spek, 2009) that in the crystal packing there are extensive O—H···O hydrogen-bond interactions (Table 1) between the O atoms of the six carboxylate/carboxylic acid groups of the H_2_TTHA^4-^ ligand and/or the water molecules, leading to a three-dimensional array (Fig. 2).

### Experimental

A mixture of Co(OH)_2_ (0.18 g, 2 mmol) and H_6_TTHA (0.20 g, 0.5 mmol) was stirred in H_2_O (30 ml) solution for 30 min at room temperature. The resulting solution was filtered and the clear solution was left standing for two weeks. Purple crystals of (I) suitable for X-ray diffraction were obtained at the bottom of the vessel.

### Refinement

C-bound H atoms were positioned geometrically (C—H = 0.97 Å) and refined with *U*_iso_= 1.2*U*_eq_(carrier atom). The carboxyl H1 and H5 atoms are each located close to a crystallographic inversion centre between pairs of symmetry equivalent atoms of O1 and O5. Both H atoms were thus refined as 50% occupied. The O—H distances were constrained to be 0.82 Å and *U*_iso_= 1.5*U*_eq_(O). Water H atoms were initially found in a difference map and refined with O—H = 0.82 Å and *U*_iso_= 1.5*U*_eq_(O). Several reflections, *i.e*. (0 0 1), (0 1 0), (2 0 0) and (-4 3 2), were omitted from the refinement owing to poor agreement.

### Crystal data

**Table:**

[Co_2_(C_18_H_26_N_4_O_12_)(H_2_O)_2_]·4H_2_O	*Z* = 1
*M**_r_* = 716.38	*F*(000) = 372
Triclinic, *P*1	*D*_x_ = 1.742 Mg m^−^^3^
Hall symbol: -P 1	Mo *K*α radiation, λ = 0.71073 Å
*a* = 7.0972 (15) Å	Cell parameters from 1506 reflections
*b* = 8.7025 (19) Å	θ = 2.6–23.8°
*c* = 11.968 (3) Å	µ = 1.31 mm^−^^1^
α = 104.238 (4)°	*T* = 292 K
β = 100.986 (3)°	Plate, violet
γ = 100.425 (4)°	0.10 × 0.08 × 0.05 mm
*V* = 682.9 (3) Å^3^	

### Data collection

**Table:**

Bruker SMART APEX CCD diffractometer	3099 independent reflections
Radiation source: fine focus sealed Siemens Mo tube	2182 reflections with *I* > 2σ(*I*)
Graphite monochromator	*R*_int_ = 0.097
0.3° wide ω exposures scans	θ_max_ = 27.5°, θ_min_ = 2.6°
Absorption correction: multi-scan (*SADABS*; Sheldrick, 2008)	*h* = −9→9
*T*_min_ = 0.881, *T*_max_ = 0.938	*k* = −11→11
8012 measured reflections	*l* = −15→15

### Refinement

**Table:**

Refinement on *F*^2^	Primary atom site location: structure-invariant direct methods
Least-squares matrix: full	Secondary atom site location: difference Fourier map
*R*[*F*^2^ > 2σ(*F*^2^)] = 0.053	Hydrogen site location: inferred from neighbouring sites
*wR*(*F*^2^) = 0.117	H-atom parameters constrained
*S* = 0.94	*w* = 1/[σ^2^(*F*_o_^2^) + (0.047*P*)^2^] where *P* = (*F*_o_^2^ + 2*F*_c_^2^)/3
3099 reflections	(Δ/σ)_max_ < 0.001
190 parameters	Δρ_max_ = 0.61 e Å^−^^3^
0 restraints	Δρ_min_ = −0.53 e Å^−^^3^

### Special details

**Table:**

Geometry. All esds (except the esd in the dihedral angle between two l.s. planes) are estimated using the full covariance matrix. The cell esds are taken into account individually in the estimation of esds in distances, angles and torsion angles; correlations between esds in cell parameters are only used when they are defined by crystal symmetry. An approximate (isotropic) treatment of cell esds is used for estimating esds involving l.s. planes.
Refinement. Refinement of F^2^ against ALL reflections. The weighted R-factor wR and goodness of fit S are based on F^2^, conventional R-factors R are based on F, with F set to zero for negative F^2^. The threshold expression of F^2^ > 2sigma(F^2^) is used only for calculating R-factors(gt) etc. and is not relevant to the choice of reflections for refinement. R-factors based on F^2^ are statistically about twice as large as those based on F, and R- factors based on ALL data will be even larger.

### Fractional atomic coordinates and isotropic or equivalent isotropic displacement parameters (Å^2^)

**Table:**

	*x*	*y*	*z*	*U*_iso_*/*U*_eq_	Occ. (<1)
Co1	0.72982 (7)	0.71114 (6)	0.70687 (4)	0.02486 (17)	
N1	0.9888 (4)	0.6394 (4)	0.6502 (2)	0.0260 (7)	
N2	0.9015 (4)	0.9540 (4)	0.7434 (2)	0.0255 (7)	
O1	1.0763 (4)	0.5057 (4)	0.9155 (2)	0.0457 (8)	
H1	1.0240	0.5153	0.9715	0.069*	0.50
O2	0.8739 (4)	0.6544 (3)	0.8584 (2)	0.0306 (6)	
O3	0.6757 (5)	0.9168 (4)	0.4387 (2)	0.0548 (9)	
O4	0.6154 (4)	0.7492 (3)	0.5486 (2)	0.0308 (6)	
O5	0.6500 (4)	1.0653 (3)	0.9745 (2)	0.0401 (7)	
H5	0.5449	1.0184	0.9830	0.060*	0.50
O6	0.5890 (4)	0.8431 (3)	0.8222 (2)	0.0335 (6)	
O7	0.5376 (4)	0.4898 (3)	0.6630 (2)	0.0416 (7)	
H7A	0.5779	0.4274	0.6978	0.062*	
H7B	0.5143	0.4317	0.5943	0.062*	
C1	0.9172 (5)	0.5213 (5)	0.5288 (3)	0.0322 (9)	
H1A	0.8342	0.5670	0.4781	0.039*	
H1B	0.8362	0.4217	0.5334	0.039*	
C2	1.0911 (6)	0.5704 (5)	0.7386 (3)	0.0395 (10)	
H2A	1.2292	0.6286	0.7657	0.047*	
H2B	1.0851	0.4570	0.7004	0.047*	
C3	1.0033 (6)	0.5803 (5)	0.8454 (3)	0.0302 (9)	
C4	1.1120 (6)	0.7947 (5)	0.6468 (3)	0.0313 (9)	
H4A	1.0630	0.8154	0.5719	0.038*	
H4B	1.2472	0.7852	0.6518	0.038*	
C5	1.1076 (6)	0.9363 (5)	0.7487 (3)	0.0351 (10)	
H5A	1.1622	0.9181	0.8237	0.042*	
H5B	1.1887	1.0362	0.7446	0.042*	
C6	0.8262 (6)	1.0170 (5)	0.6438 (3)	0.0362 (10)	
H6A	0.9377	1.0745	0.6224	0.043*	
H6B	0.7507	1.0950	0.6706	0.043*	
C7	0.6972 (6)	0.8852 (5)	0.5344 (3)	0.0330 (9)	
C8	0.8807 (6)	1.0525 (5)	0.8581 (3)	0.0310 (9)	
H8A	0.8829	1.1632	0.8554	0.037*	
H8B	0.9912	1.0565	0.9215	0.037*	
C9	0.6896 (6)	0.9799 (5)	0.8838 (3)	0.0284 (8)	
O8	0.5622 (5)	0.2722 (4)	0.7785 (3)	0.0729 (11)	
H8C	0.5855	0.3176	0.8501	0.109*	
H8D	0.4441	0.2302	0.7658	0.109*	
O9	0.3797 (6)	0.3141 (5)	0.9602 (4)	0.1021 (15)	
H9A	0.3722	0.2969	1.0237	0.153*	
H9B	0.2845	0.3520	0.9418	0.153*	

### Atomic displacement parameters (Å^2^)

**Table:**

	*U*^11^	*U*^22^	*U*^33^	*U*^12^	*U*^13^	*U*^23^
Co1	0.0255 (3)	0.0292 (3)	0.0196 (3)	0.0075 (2)	0.0068 (2)	0.0049 (2)
N1	0.0287 (17)	0.0341 (18)	0.0182 (15)	0.0108 (14)	0.0098 (13)	0.0076 (13)
N2	0.0281 (17)	0.0298 (17)	0.0190 (15)	0.0065 (14)	0.0094 (13)	0.0057 (13)
O1	0.055 (2)	0.068 (2)	0.0344 (16)	0.0356 (17)	0.0201 (15)	0.0306 (16)
O2	0.0338 (15)	0.0446 (17)	0.0198 (13)	0.0194 (13)	0.0112 (12)	0.0102 (12)
O3	0.077 (2)	0.055 (2)	0.0289 (16)	0.0015 (17)	0.0066 (16)	0.0207 (15)
O4	0.0358 (16)	0.0321 (15)	0.0222 (13)	0.0067 (12)	0.0031 (12)	0.0077 (12)
O5	0.0412 (17)	0.0452 (18)	0.0286 (15)	0.0088 (14)	0.0185 (13)	−0.0050 (13)
O6	0.0301 (15)	0.0372 (16)	0.0306 (15)	0.0082 (13)	0.0126 (12)	0.0010 (13)
O7	0.0489 (18)	0.0369 (17)	0.0311 (16)	0.0011 (14)	0.0062 (14)	0.0051 (13)
C1	0.033 (2)	0.034 (2)	0.030 (2)	0.0091 (18)	0.0158 (18)	0.0037 (18)
C2	0.045 (3)	0.057 (3)	0.031 (2)	0.029 (2)	0.018 (2)	0.021 (2)
C3	0.031 (2)	0.037 (2)	0.0220 (19)	0.0077 (18)	0.0068 (17)	0.0078 (17)
C4	0.027 (2)	0.035 (2)	0.031 (2)	0.0057 (17)	0.0105 (17)	0.0063 (18)
C5	0.027 (2)	0.039 (2)	0.033 (2)	−0.0019 (18)	0.0085 (18)	0.0038 (19)
C6	0.051 (3)	0.032 (2)	0.026 (2)	0.0106 (19)	0.0110 (19)	0.0079 (18)
C7	0.036 (2)	0.041 (2)	0.025 (2)	0.0128 (19)	0.0092 (18)	0.0110 (18)
C8	0.036 (2)	0.032 (2)	0.0201 (19)	0.0051 (18)	0.0072 (17)	0.0009 (16)
C9	0.030 (2)	0.033 (2)	0.0223 (19)	0.0117 (18)	0.0046 (17)	0.0078 (17)
O8	0.073 (3)	0.077 (3)	0.064 (2)	0.005 (2)	0.010 (2)	0.027 (2)
O9	0.121 (4)	0.106 (4)	0.115 (4)	0.058 (3)	0.069 (3)	0.043 (3)

### Geometric parameters (Å, º)

**Table:**

Co1—O7	2.031 (3)	C1—C1^i^	1.527 (7)
Co1—O4	2.043 (2)	C1—H1A	0.9700
Co1—O6	2.094 (2)	C1—H1B	0.9700
Co1—O2	2.120 (2)	C2—C3	1.516 (5)
Co1—N2	2.134 (3)	C2—H2A	0.9700
Co1—N1	2.216 (3)	C2—H2B	0.9700
N1—C2	1.477 (4)	C4—C5	1.515 (5)
N1—C4	1.486 (4)	C4—H4A	0.9700
N1—C1	1.490 (4)	C4—H4B	0.9700
N2—C8	1.478 (4)	C5—H5A	0.9700
N2—C6	1.480 (4)	C5—H5B	0.9700
N2—C5	1.490 (5)	C6—C7	1.514 (5)
O1—C3	1.272 (4)	C6—H6A	0.9700
O1—H1	0.8200	C6—H6B	0.9700
O2—C3	1.226 (4)	C8—C9	1.509 (5)
O3—C7	1.230 (4)	C8—H8A	0.9700
O4—C7	1.286 (4)	C8—H8B	0.9700
O5—C9	1.267 (4)	O8—H8D	0.8200
O5—H5	0.8200	O8—H8C	0.8200
O6—C9	1.237 (4)	O8—H8D	0.8200
O7—H7A	0.8201	O9—H9A	0.8200
O7—H7B	0.8200	O9—H9B	0.8200
			
O7—Co1—O4	92.28 (11)	N1—C2—H2A	108.9
O7—Co1—O6	97.80 (11)	C3—C2—H2A	108.9
O4—Co1—O6	102.38 (10)	N1—C2—H2B	108.9
O7—Co1—O2	87.25 (11)	C3—C2—H2B	108.9
O4—Co1—O2	172.02 (10)	H2A—C2—H2B	107.7
O6—Co1—O2	85.56 (10)	O2—C3—O1	125.6 (3)
O7—Co1—N2	172.89 (11)	O2—C3—C2	120.8 (3)
O4—Co1—N2	82.40 (11)	O1—C3—C2	113.6 (3)
O6—Co1—N2	78.85 (11)	N1—C4—C5	111.0 (3)
O2—Co1—N2	98.68 (11)	N1—C4—H4A	109.4
O7—Co1—N1	100.97 (11)	C5—C4—H4A	109.4
O4—Co1—N1	93.72 (10)	N1—C4—H4B	109.4
O6—Co1—N1	154.68 (11)	C5—C4—H4B	109.4
O2—Co1—N1	78.57 (10)	H4A—C4—H4B	108.0
N2—Co1—N1	84.15 (11)	N2—C5—C4	110.7 (3)
C2—N1—C4	112.2 (3)	N2—C5—H5A	109.5
C2—N1—C1	112.8 (3)	C4—C5—H5A	109.5
C4—N1—C1	110.5 (3)	N2—C5—H5B	109.5
C2—N1—Co1	108.9 (2)	C4—C5—H5B	109.5
C4—N1—Co1	103.8 (2)	H5A—C5—H5B	108.1
C1—N1—Co1	108.1 (2)	N2—C6—C7	113.7 (3)
C8—N2—C6	111.9 (3)	N2—C6—H6A	108.8
C8—N2—C5	112.3 (3)	C7—C6—H6A	108.8
C6—N2—C5	111.7 (3)	N2—C6—H6B	108.8
C8—N2—Co1	108.2 (2)	C7—C6—H6B	108.8
C6—N2—Co1	107.5 (2)	H6A—C6—H6B	107.7
C5—N2—Co1	104.7 (2)	O3—C7—O4	124.8 (4)
C3—O1—H1	109.5	O3—C7—C6	117.7 (4)
C3—O2—Co1	116.4 (2)	O4—C7—C6	117.5 (3)
C7—O4—Co1	115.5 (2)	N2—C8—C9	110.6 (3)
C9—O5—H5	109.5	N2—C8—H8A	109.5
C9—O6—Co1	114.6 (2)	C9—C8—H8A	109.5
Co1—O7—H7A	113.6	N2—C8—H8B	109.5
Co1—O7—H7B	117.3	C9—C8—H8B	109.5
H7A—O7—H7B	99.1	H8A—C8—H8B	108.1
N1—C1—C1^i^	113.9 (4)	O6—C9—O5	124.5 (4)
N1—C1—H1A	108.8	O6—C9—C8	120.5 (3)
C1^i^—C1—H1A	108.8	O5—C9—C8	114.9 (3)
N1—C1—H1B	108.8	H8D—O8—H8C	99.3
C1^i^—C1—H1B	108.8	H8C—O8—H8D	99.3
H1A—C1—H1B	107.7	H9A—O9—H9B	104.7
N1—C2—C3	113.2 (3)		
			
O7—Co1—N1—C2	−76.1 (3)	O4—Co1—O6—C9	−99.2 (3)
O4—Co1—N1—C2	−169.1 (2)	O2—Co1—O6—C9	80.1 (3)
O6—Co1—N1—C2	61.1 (4)	N2—Co1—O6—C9	−19.6 (3)
O2—Co1—N1—C2	8.8 (2)	N1—Co1—O6—C9	29.1 (4)
N2—Co1—N1—C2	108.9 (3)	C2—N1—C1—C1^i^	−67.4 (5)
O7—Co1—N1—C4	164.2 (2)	C4—N1—C1—C1^i^	59.1 (5)
O4—Co1—N1—C4	71.1 (2)	Co1—N1—C1—C1^i^	172.2 (4)
O6—Co1—N1—C4	−58.6 (3)	C4—N1—C2—C3	109.8 (4)
O2—Co1—N1—C4	−110.9 (2)	C1—N1—C2—C3	−124.6 (3)
N2—Co1—N1—C4	−10.8 (2)	Co1—N1—C2—C3	−4.6 (4)
O7—Co1—N1—C1	46.8 (2)	Co1—O2—C3—O1	−164.1 (3)
O4—Co1—N1—C1	−46.3 (2)	Co1—O2—C3—C2	15.6 (5)
O6—Co1—N1—C1	−176.0 (2)	N1—C2—C3—O2	−6.9 (5)
O2—Co1—N1—C1	131.7 (2)	N1—C2—C3—O1	172.8 (3)
N2—Co1—N1—C1	−128.2 (2)	C2—N1—C4—C5	−79.2 (4)
O4—Co1—N2—C8	127.7 (2)	C1—N1—C4—C5	154.0 (3)
O6—Co1—N2—C8	23.4 (2)	Co1—N1—C4—C5	38.2 (3)
O2—Co1—N2—C8	−60.3 (2)	C8—N2—C5—C4	161.8 (3)
N1—Co1—N2—C8	−137.7 (2)	C6—N2—C5—C4	−71.5 (4)
O4—Co1—N2—C6	6.6 (2)	Co1—N2—C5—C4	44.6 (3)
O6—Co1—N2—C6	−97.7 (2)	N1—C4—C5—N2	−59.1 (4)
O2—Co1—N2—C6	178.6 (2)	C8—N2—C6—C7	−134.5 (3)
N1—Co1—N2—C6	101.2 (2)	C5—N2—C6—C7	98.6 (4)
O4—Co1—N2—C5	−112.3 (2)	Co1—N2—C6—C7	−15.7 (4)
O6—Co1—N2—C5	143.4 (2)	Co1—O4—C7—O3	165.6 (3)
O2—Co1—N2—C5	59.7 (2)	Co1—O4—C7—C6	−15.6 (4)
N1—Co1—N2—C5	−17.8 (2)	N2—C6—C7—O3	−159.4 (4)
O7—Co1—O2—C3	88.1 (3)	N2—C6—C7—O4	21.6 (5)
O6—Co1—O2—C3	−173.8 (3)	C6—N2—C8—C9	93.7 (4)
N2—Co1—O2—C3	−95.8 (3)	C5—N2—C8—C9	−139.8 (3)
N1—Co1—O2—C3	−13.7 (3)	Co1—N2—C8—C9	−24.7 (3)
O7—Co1—O4—C7	−179.9 (3)	Co1—O6—C9—O5	−165.2 (3)
O6—Co1—O4—C7	81.6 (3)	Co1—O6—C9—C8	10.9 (4)
N2—Co1—O4—C7	4.8 (3)	N2—C8—C9—O6	10.2 (5)
N1—Co1—O4—C7	−78.8 (3)	N2—C8—C9—O5	−173.3 (3)
O7—Co1—O6—C9	166.7 (3)		

Symmetry code: (i) −*x*+2, −*y*+1, −*z*+1.

### Hydrogen-bond geometry (Å, º)

**Table:**

*D*—H···*A*	*D*—H	H···*A*	*D*···*A*	*D*—H···*A*
O1—H1···O1^ii^	0.82	1.68	2.483 (5)	168
O5—H5···O5^iii^	0.82	1.67	2.481 (5)	170
O7—H7*A*···O8	0.82	1.84	2.616 (4)	157
O7—H7*B*···O4^iv^	0.82	1.95	2.732 (4)	159
O8—H8*D*···O3^iv^	0.82	2.37	2.745 (5)	109
O8—H8*C*···O9	0.82	2.15	2.719 (5)	127
O9—H9*A*···O2^v^	0.82	2.48	3.067 (4)	130
O9—H9*A*···O6^v^	0.82	2.45	3.217 (5)	157

Symmetry codes: (ii) −*x*+2, −*y*+1, −*z*+2; (iii) −*x*+1, −*y*+2, −*z*+2; (iv) −*x*+1, −*y*+1, −*z*+1; (v) −*x*+1, −*y*+1, −*z*+2.
